# The 5-Lipoxygenase as a Common Pathway for Pathological Brain and Vascular Aging

**DOI:** 10.1155/2009/174657

**Published:** 2009-09-01

**Authors:** Jin Chu, Domenico Praticò

**Affiliations:** Department of Pharmacology School of Medicine, Temple University, Philadelphia, PA 19140, USA

## Abstract

Epidemiological studies indicate age as a strong risk factor for developing cardiovascular and neurodegenerative diseases. During the aging process, changes in the expression of particular genes can influence the susceptibility to these diseases. 5-Lipoxygenase (5-LO) by oxidizing fatty acids forms leukotrienes, potent mediators of oxidative and inflammatory reactions, two key pathogenic events in both clinical settings. This enzyme is widely distributed in the cardiovascular as well as in the central nervous system, where its expression levels increase with age, suggesting that it may be involved in their diseases of aging. The central theme of this article is that during aging, 5-LO acts as biologic link between different stressors and the development of cardiovascular and neurodegenerative diseases. We hypothesize that the age-dependent upregulation of 5-LO represents a “priming” factor in the vasculature as well as in the brain, where a subsequent exposure to triggering stimuli (i.e., infections) leads to an abnormal chronic inflammatory reaction, and ultimately results in increased organ vulnerability and functional deficits.

## 1. Introduction

Consistent demographic data show that due to the improvements in public health and advances in medical therapy, the number of older people (over 65 years) is fast increasing worldwide, and it is expected to triple by 2040. 

Since advancing age is the strongest risk factor for developing chronic diseases, the burden from them is expected to increase several-fold over the next 15–20 years. This fact has created a sense of emergency toward this segment of the population in view of the potential catastrophic socioeconomic consequences. Interestingly, age is a nonmodifiable risk factor for atherosclerosis and chronic neurodegenerative diseases such as Alzheimer's disease (AD) [1, 2]. The aging process is the most common feature of the postreproductive phase of life. It manifests in all multicellular organisms and is characterized by a progressive reduction in the efficacy of a number of physiological processes. This decline translates to a reduced capacity to maintain homeostatic control of important functions and finally results in increased organ vulnerability. 

In experimental models, for example, aged animals have an exacerbation to experimental vascular injury and develop atherosclerosis even on a chow diet [3]. On the other hand, they also exhibit an impaired ability to sustain long-term potentiation, a form of synaptic plasticity that has been proposed as biological substrate for learning and/or memory [4], and have impaired spatial learning in the Morris water maze [5].

## 2. The 5-LO Pathway in the Vasculature and Central Nervous System

**Figure 1:**
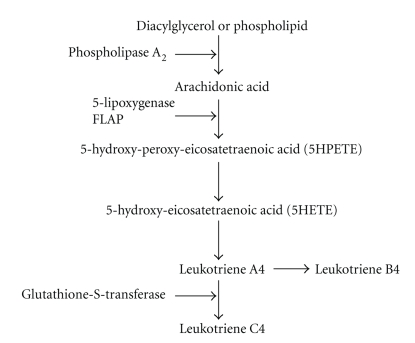
Schematic representation of the 5-Lipoxygenase enzyme metabolic pathway. Arachidonic acid is released from diacyglycerol or membrane phospholipids via the action of Phospholipase A_2_. Once free, arachidonic acid is oxidized by 5-lipoxygenase (5-LO), which has been activated by the Five-Lipoxygenase-Activating-Protein (FLAP), at carbon 5 to form the unstable 5-hydroxy-peroxy-eicosatetraenoic acid (5HPETE), which is promptly metabolized into the more stable 5-hydroxy-eicosatetraneoic acid (5HETE). The 5HETE can then be converted in leukotriene A4 (LTA4), which can serve either as an intracellular intermediate in the synthesis of LTB4 and LTC4, or may be released extracellularly and subsequently be taken up by adjacent cells devoid of 5-LO activity but expressing LTA4-hydrolase and/or LTC4 synthase.

5-Lipoxygenase (5-LO) is a member of a large family of enzymes, called lipoxygenases, which oxidizes free and esterified polyunsaturated fatty acids. 5-LO first introduces active molecular oxygen to carbon 5 of arachidonic acid resulting in the formation of 5-Hydroxy-peroxy-eicosatetraenoic acid (5HPETE). This unstable derivative is either reduced to 5-Hydroxy-eicosatetraenoic acid (5HETE), or converted to leukotriene (LT) A4. However, LTA4 can serve either as an intracellular intermediate in the synthesis of LTB4 and LTC4, or may be released extracellularly and subsequently be taken up by adjacent cells devoid of 5-LO activity but expressing LTA4-hydrolase and/or LTC4 synthase. LTs and the cysteinyl derivatives of LTs all have strong pro-oxidant and proinflammatory activities [6] (see Figure 1). 

5-LO is widely expressed in the cardiovascular system, that is, aorta, coronary, and carotid arteries, as well as in macrophages and neutrophils. Interestingly, its expression levels are increased in aortas of old animals when compared with young ones [7]. This enzymatic pathway is also widely expressed in the central nervous system (CNS), where it localizes mainly in neuronal cells of the hippocampus and cortex, and, similar to the vasculature, its levels increase significantly with aging [8, 9]. 

The expression of 5-LO is susceptible to hormonal regulation, since higher levels are observed in conditions of melatonin deficiency and/or hyperglucocorticoidemia [10, 11], both of which are common in elderly subjects [12]. Although in general upregulation of 5-LO might serve a physiological purpose, during the aging process, it may also increase the vulnerability of the cardiovascular system and CNS to different insults/stressors [13]. Given that older subjects are at greater risk of health complications and mortality stemming from altered inflammatory and immune functions, and aging, via the upregulation of 5-LO, can be an important risk factor, the effects of stressors on this enzymatic pathway are of particular importance.

## 3. 5-LO, Aging and Cardiovascular Diseases

Recent studies have implicated 5-LO in the pathogenesis of atherosclerosis [14], and have also identified specific 5-LO genotypes in subpopulations with increased risk of atherosclerosis [15, 16]. 

Age is an established risk factor for atherosclerosis. Among primates and rodents, older animals develop more extensive atherosclerosis than younger animals [17, 18]. Age-accelerated vascular injury is commonly considered to result from increased oxidative stress, leading to inflammation and endothelial dysfunction [19]. Tissues from aged animals demonstrate increase generation of reactive oxygen species (ROSs) that lead to damage to vascular cells with age-associated remodeling changes, and oxidation of lipids, that is, leukotrienes, with potent proinflammatory and proatherogenic actions [20, 21]. Interestingly, in experimental models of atherogenesis, the disease process can be exacerbated by inflammatory stress such as lipopolysaccharide (LPS) exposure [22–24]. LPS binds to the Toll-like receptor 4 (TLR4) on the surface of a variety of cell types stimulating, among other things, the generation of inflammatory leukotrienes derived from the 5-LO pathway [25]. In this setting, pharmacologic blockade of this enzyme or its genetic deficiency affords a significant protective effect against organ injury and dysfunction [26]. These facts, together with the upregulation of 5-LO in the aging vasculature support the hypothesis that this enzymatic pathway plays a functional role in the development of aging-related cardiovascular diseases.

## 4. 5-LO, Aging and Neurodegenerative Diseases

In the CNS, aging, in general, is associated with an increased incidence of chronic neurodegenerative processes, and among them, AD is the most frequent [27, 28]. From a biochemical point of view, brain aging is often associated with microglia activation and a diffuse and chronic brain inflammation involving also other cell types, that is, neurons [29]. Interestingly, aged animals show greater increase in central inflammatory cytokines compared with young adults following both peripheral and central LPS administration [30, 31]. This response is accompanied by a greater deficit in spatial working memory than is seen in young adult mice [32, 33], and suggests a possible modulatory role for 5-LO. Thus, as stress appears to sensitize the CNS to subsequent insults, so may aging with the upregulation of 5-LO sensitizes cells of the immune system to stress itself. Although there is evidence that stress can influence immune responses and memory performance in elderly, however, the direct effect of 5-LO on these phenomena is still largely unknown. In the aging brain, the prolonged stress-dependent inflammation status could then serve as precursor for augmented neuronal vulnerability which often culminates in cell death and loss of function. Beside the inflammatory hypothesis, recent work has also highlighted a novel concept that 5-LO can directly modulate neurotransmitter receptors as well as amyloid beta peptide metabolism, both of which are well-established mechanisms involved in brain aging [34, 35].

## 5. Peripheral Stressors: Effect on Cardiovascular and Neurodegenerative Diseases

**Figure 2:**
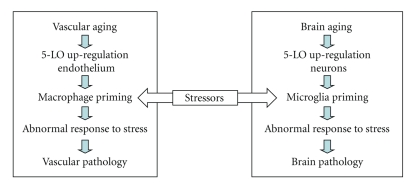
Hypothetical model whereby 5-Lipoxygenase influences brain and vascular pathological aging. During aging, peripheral and central stressors targeting the vasculature and/or the central nervous system find these organs primed to a chronic inflammatory status secondary to the upregulation of 5-LO in endothelial cells and macrophages, neurons, and microglia, respectively. This fact facilitates an abnormal and long-lasting inflammatory response, which ultimately results in increased organ vulnerability, functional impairments, and development of pathology.

Stress is a risk factor for pathological aging because elderly individuals prone to psychological distress are more likely to develop cardiovascular and/or neurodegenerative disorders than age-matched controls, nonstressed individuals. Recent studies suggest that activation of peripheral immune system elicits a discordant inflammatory response in aged but, otherwise, healthy subjects compared with younger cohorts, and the reactive state of immune cells in the aged individuals has been suggested as the basis for this abnormal inflammatory response. We hypothesize that the upregulation of 5-LO in the aging brain and vasculature by releasing high amount of leukotrienes functions as priming factor for these organs facilitating an abnormal inflammatory response to stressors, which ultimately results in increased organ vulnerability and functional impairments (Figure 2). 

Importantly, while these responses are transient and reversible in young, by contrast they are generally exacerbated and long-lasting in aged subjects. 

In what follows, we briefly discuss two models of stress which have been widely used in the aging field. Both of them mimick in vivo biologically relevant situations: LPS, as bacterial infection (very frequent in elderly); high levels of glucocorticosteroids (as it is typically observed in aging) [36, 37].

## 6. LPS

Administration of LPS has been widely used as a model to trigger both vascular and neuroinflammatory responses [38–41]. These inflammatory responses, in part mediated by 5-LO activation, could act in concert with aging to accelerate vascular and neuronal vulnerability and subsequent cell loss. It is conceivable that the 5-LO upregulation in endothelial cells and neurons, typical of the aging process, can function as priming event toward macrophages/microglia in these systems and sensitizes them to an increased susceptibility and abnormal biological response to stressors (Figure 2).

## 7. Glucocorticoids

Recent data suggest that glucocorticoid-sensitive mechanism(s) are operative in increasing neuronal vulnerability of the aging brain. Thus, high glucocorticoid levels appear to be a constant feature of senile dementia [42]. Aged humans with prolonged elevated levels of cortisol exhibit reduced hippocampal volume and deficits in hippocampus-dependent memory tasks compared with normal cortisol controls. Further, glucocorticoids can negatively affect neuronal survival [43] in vitro, and impairs cognition in vivo [44]. Similarly, a dysregulated cortisol secretion, may be secondary to abnormalities in the hypothalamic-pituitary-adrenal axis, has also been involved in the failure to contain inflammatory reactions within the vasculature [45, 46]. In both scenarios, the age-dependent 5-LO upregulation could further sensitize these organs to glucocorticoid-mediated detrimental effects (Figure 2). 

In both cases, the hypothesis could be easily tested considering the availability of different selective inhibitors of this enzymatic pathway, together with mice which are genetically deficient for this enzyme. Thus, treating aged animals with LPS or corticosteroids in the presence of the inhibitors, or aged 5-LO knock-out and wild-type mice with the same stressors could provide us with important information supporting the functional role of this metabolic pathway.

## 8. Conclusions

Because of the projected aging of the human population, the burden from diseases of aging is expected to increase dramatically over the next 20–25 years. The identification of a putative common molecular mechanism influencing these diseases and amenable of a therapeutic modulation would result not only in an improvement of the quality of life for this segment of the population but also in a significantly reduced socio-economic impact of these diseases. The fact that the 5-LO is significantly increased with aging, which associates with the development of cardiovascular as well as neurodegenerative diseases, makes this enzymatic pathway an excellent candidate that fulfills these criteria.

Several molecular mechanisms have been invoked for the 5-LO-mediated age-dependent increased cardiovascular risk, and most of them involve modulation of the inflammatory vascular response to stressors. By contrast, much less is known about the molecular mechanisms operating in the 5-LO-mediated pathologic brain aging. Beside the role of 5-LO in regulating neuroinflammation, more recent works have pointed out some novel mechanisms and pathways whereby this enzyme may be involved in pathological brain aging, that is, neurotransmitter receptors and amyloid beta peptide metabolism. 

Future studies are warranted to provide a more conclusive evidence for this association, and new or revisited common molecular mechanisms responsible for it.
